# Truth-telling and doctor-assisted death as perceived by Israeli physicians

**DOI:** 10.1186/s12910-019-0350-5

**Published:** 2019-02-18

**Authors:** Baruch Velan, Arnona Ziv, Giora Kaplan, Carmit Rubin, Yaron Connelly, Tami Karni, Orna Tal

**Affiliations:** 1 TheGertner Institute for Epidemiology and Health Policy Research, Sheba Medical Center, Ramat Gan, Israel; 2 Israeli Center for Emerging Technologies, Shamir Medical Center (Assaf Harofeh), Zerifin, Israel; 3 Comprehensive Breast Care Institute, Shamir Medical Center (Assaf Harofeh), Zerifin, Israel

**Keywords:** End-of-life, Doctor-assisted-death, Disclosure, Truth-telling, Autonomy, Ethical education

## Abstract

**Background:**

Medicine has undergone substantial changes in the way medical dilemmas are being dealt with. Here we explore the attitude of Israeli physicians to two debatable dilemmas: disclosing the full truth to patients about a poor medical prognosis, and assisting terminally ill patients in ending their lives.

**Methods:**

Attitudes towards medico-ethical dilemmas were examined through a nationwide online survey conducted among members of the Israeli Medical Association, yielding 2926 responses.

**Results:**

Close to 60% of the respondents supported doctor-assisted death, while one third rejected it. Half of the respondents opposed disclosure of the full truth about a poor medical prognosis, and the others supported it. Support for truth-telling was higher among younger physicians, and support for doctor-assisted death was higher among females and among physicians practicing in hospitals. One quarter of respondents supported both truth-telling and assisted death, thereby exhibiting respect for patients’ autonomy. This approach characterizes younger doctors and is less frequent among general practitioners. Another quarter of the respondents rejected truth-telling, yet supported assisted death, thereby manifesting compassionate pragmatism. This was associated with medical education, being more frequent among doctors educated in Israel, than those educated abroad. All this suggests that both personal attributes and professional experience affect attitudes of physicians to ethical questions.

**Conclusions:**

Examination of attitudes to two debatable medical dilemmas allowed portrayal of the multi-faceted medico-ethical scene in Israel. Moreover, this study, demonstrates that one can probe the ethical atmosphere of a given medical community, at various time points by using a few carefully selected questions.

**Electronic supplementary material:**

The online version of this article (10.1186/s12910-019-0350-5) contains supplementary material, which is available to authorized users.

## Background

Medicine has undergone substantial transformations in recent decades. This is manifested not only by major technological advances allowing the provision of better healthcare, but also by conceptual changes which have significantly altered the nature of medical practice.

One major shift has been the rise of focus on medical ethics. The ongoing technological and social changes have sparked growth in the philosophical debate over problems pertaining to medical practice. The emergences of new moral dilemmas that can no longer be found in the Hippocrates’ ethical model have led to a paradigm shift in which a set of ethical principles is used to make complex medical decisions. The bioethics movement, which has boomed during the last decades of the twentieth century, has engendered different ethical theories, and brought to focus principlism as a leading approach for solving problems in clinical practice. Principlism defines the common ground moral principles of beneficence, nonmaleficence, justice and, autonomy, underlining, among others, patient capacity to think, decide and act on his/her own free initiative [1].

Another shift in medical practice relates to redefinitions of the interactions between physicians and patients. This has been affected not only by the substantial attention given to patient autonomy, but also by the fact that patients are now better informed [2]. The internet and social media have accelerated the patient’s ability and willingness to take part in medical decisions [3] and at the same time litigation has become more common. In addition, ongoing developments are continuously challenging the traditional definitions of medicine. The core definition of medical work as attention to ‘disease’ and “‘illnesses” does not seem to suffice in describing the broad spectrum of activities subsumed under the umbrella of medicine [4, 5]. In addition, the definitions of the physicians’ involvement in the beginning of life and the end of life became broader [6].

All these developments have led to notable changes in the way major medical dilemmas are being dealt with. Among these are dilemmas related to telling the full truth to patients about poor medical prognosis, as well as questions about the legitimacy of doctor assistance in terminating the lives of terminally ill patients upon their request.

Attitudes to truth-telling in medicine can serve as an effective tool to monitor changes in medical ethics. While truth-telling is widely affirmed as a cardinal moral obligation, deception in medicine was considered legitimate up to the second half of the twentieth century [7]. Disclosure of full medical information to patients has moved from being considered a harmful practice at the beginning of the twentieth century [8], to become an inherent duty at the end of the century [9]. Nevertheless, the debate over truth-telling has not been settled, and continues to stir medical communities. This debate is ruled by three conflicting approaches: the consequential approach, which assesses truth-telling by the benefits and harms that are entailed [10, 11]; the non-consequential approach, which relates to truth-telling as a categorical imperative [12], or as an essential component of patient autonomy [13]; and the third approach, which is gaining renewed interest, addresses truth-telling in a relativistic, culture-dependent manner, and suggests that truth should be tailored differently to different patients [14]. All this becomes even more complicated in the patient-doctor relationship, since truth remains particular, ambiguous, provisional, and dynamic [15].

The attitudes of the medical community to end of life issues can also serve as a tool for monitoring changes in medical ethics. While, death inflicted by a physician would have been morally unconceivable in the years after WWII, a major change has occurred in the last decades of the twentieth century. The debate over the role of doctors in helping patients die, when this becomes the last resort option has become part of the ethical discourse [16]. Such help can take various forms. Physicians can stop life-sustaining therapy, such as mechanical ventilation, renal dialysis, or tube feeding [17]. Physicians can accelerate opioid-treatment for severe pain management to levels that lead to unconsciousness and eventually death [18]. Physicians can participate in doctor-assisted death (called also physician-assisted suicide), where a physician provides, at the patient’s request, the means, and the patient must carry out the final act [19]. Finally, physicians can also practice voluntary active euthanasia, where a physician provides the means and carries out the final act [20]. All these forms of assisted dying are obviously subjected to the presence of volunteerism and free will.

The moral argumentation used by the supporters of assisted death, invokes compassion and beneficence, as well as a high respect for patient autonomy. Rejection of such practices is based on arguments related to deontology (one should not kill), fear of slippery slopes, maleficence and the belief that life termination is not part of the set of duties that physicians should abide by [21, 22].

Various forms of assisted death are now legalized in several western countries. Euthanasia by physicians was first allowed in the Netherlands by judicial fiat in 1973. After three decades of debate, the Dutch parliament decided that euthanasia should be legalized. The act of 2002 allowed patients meeting several criteria to qualify for euthanasia and protected doctors from prosecution [23]. Belgium allowed euthanasia in 2002, based on criteria similar to those of the Dutch law [24]. In the US, the state of Oregon allowed assisted dying in 1997, and was followed by Washington, Vermont, Montana, California, Colorado, Hawaii and DC [25]. In Canada, physician-assisted suicide has been legal in the Province of Quebec since 2014, and became legal in the entire country in 2015 [26]. Debates about legalizing assisted death have been taking place in many countries [27], but have not led to actual legalization. Therefore, the question about a widespread “exportability” of the Dutch/Belgian model remains open.

In Israel the debate about assisted dying among scholars began immediately after the 2002 Dutch ruling [28] and has been going on since then with varying intensities [29, 30]. In 2014, public debates were intensified when a proposed bill for legalizing physician assisted suicide passed a Ministerial Committee. Nevertheless, the law failed to come into being because of strong opposition from conservative legislators and religious leaders. At present, the right to die with dignity in Israel is regulated according to the provisions of the law, which establishes that in certain situations, someone defined as a terminally ill patient is entitled to request a cessation of medical treatment, and the medical staff is obligated to comply with the patient’s wishes [30]. Any other assistance with life termination carries a penalty of up to 20 years of imprisonment.

In this study we have examined the attitude of Israeli physicians to the two fundamental dilemmas discussed above: Should doctors tell the full truth (TFT) to their patients when the final prognosis is poor, and should physicians take part in doctor-assisted death (DAD). The attitude of medical communities to these two dilemmas can reflect the major changes in medical ethics occurring during the last four decades. In addition, mapping the attitudes to these two dilemmas can monitor the ongoing tension between deontological ethics and utilitarian ethics as expressed in practical medical ethics: While perceptions of truth-telling in medicine have moved in recent decades from ethical pragmatism to a fundamental command, the trajectory of the perception of assisted dying has been the reverse, moving from fundamental morality to ethical pragmatism.

In this study we examine the attitudes of Israeli physicians to dilemmas related to truth-telling and assisted dying in an attempt to monitor the ethical atmosphere in the medical community in this country. We focus on Israel, as an example of a multicultural society, where both, traditional along with very modern approaches can affect moral perceptions, as well as medical practices.

## Methods

### Survey

Registered members of the Israeli Medical Association (IMA) were invited by the chair of the IMA’s ethics bureau to take an online survey aimed at evaluating ethical attitudes among Israeli physicians. The survey was distributed via email. The physicians’ responses were voluntary and anonymous. The survey was sent to 29.208 IMA members with eligible E-mail addresses (out of 32.281 members who provided contact addresses and personal information upon registration). The survey was sent on the 22 of January 2018, followed by three reminders. 2926 physicians completed the questionnaire, 1214 of them following the initial request and 1712 more following the reminders. Overall 10% of IMA members with contact addresses responded to the questionnaire.

Physicians were asked to answer 8 questions related to different ethical dilemmas encountered in medical practice. In this article we shall refer only to two of the questions, one related to telling the truth to patients about a poor prognosis, and the other related to assisting patients to die. The other questions related to issues such as prescription of highly expensive drugs, administration of futile treatments, breaching of medical secrecy, disclosure of malpractices, or compliance with institutional directives. All these will be treated in a different article as they do not compare to the very fundamental ethical dilemmas of life termination and truth-telling.

The question relating to truth-telling was phrased as following: “Should the physician tell a patient the truth about his/her medical condition in cases of a very poor prognosis?” Three options for answers were proposed. #1) Physicians are required to tell the patient the full truth, since the patient has the right to know; #2) Physicians are not required to tell the truth if they believe that this will harm patient; #3) I am unable to choose between the two options. The question relating to assisted death was phrased as following: “Would you assist a fatally ill patient in terminating his life if his medical condition justified it, and given that this is compatible with the Law?” Three options for answers were proposed. #1) I would do it, since a doctor has to assist his patients even in the final stages; #2) I would not do it, since physicians should not collaborate with life termination #3) I am unable to choose between the two options.

The survey also included a set of questions aimed at recording the respondent’s personal and professional characteristics. These questions were phrased as following: “Please state your gender”; “Please state your year of birth”; “Where did you acquire your medical education” (choose between Israel, abroad, or combined). What is your position (Choose between intern, specialist, super-specialist and other). “Please state your major workplace” (choose between HMO, public hospital, private clinic, private hospital, the military and retirement). Please define your field of expertise (self definition).

### Statistical analysis

Statistical analysis was performed using the chi-square test for comparison of attitudes between subgroups of categorical variables. A *p*-value of less than 0.05 was considered statistically significant. Data were weighted according to the actual distribution of age gender and specialization of IMA members (Table 1). Results presented in Table 1 and in the supplementary tables represent actual survey data, yet weighted data are provided thought the text. The weighted logistic regression for multivariate analysis was used to adjust attitudes to TFT and DAD for possible.

**Table 1:** Representation of sub-groups in the survey population and in the Israel Medical Association population

Population characteristics		Representation in survey populations ((*n* = 2926) %	Representation in IMA populations ((*n*=) %
Gender	Male	64.8	60.2
	Female	35.2	39.8
Age	24–34	11.5	20.4
	35–44	20.8	22.2
	45–54	20.0	19.3
	55–64	25.9	19.1
	65+	21.8	18.2
	Unknown	0.02	0.9
Specialization	GPs	14.2	8.8
	Internists	28.4	26.4
	Surgeons	21.4	15.4
	Pediatricians	13.5	11.7
	Gynecologists	7.3	6.0
	Psychiatrist	6.7	4.5
	Others	8.5	11.0
	Unknown	0.06	16.2
Medical Schooling	Israel	51.1	No Data
	Abroad	24.9	No Data
	Combined	24.0	No Data
Position	Interns	16.0	No Data
	Specialists	45.1	No Data
	Super specialists	30.3	No Data
	Other	8.6	No Data
Employment	HMOs	29.6	No Data
	Public Hospitals	49.4	No Data
	Other	21.0	No Data

socio-demographic variables. Analysis was performed using SAS statistical procedures (SAS Institute, Cary, NC, USA).

## Results

### Participants’ characteristics

Respondent characteristics are presented in Table 1. 35.2% of the respondents identified themselves as females. The mean age of respondents was 52.8 + 14.0, and the various age groups were well represented. Out of the respondents 51% were educated in Israeli medical schools, 25% attended medical schools abroad, and 24% experienced a combined medical education, in Israel and abroad.

Out of the respondents 28.4% identified themselves as internists, 21.4% as surgeons, 14.2% as general practitioners, 13.5% as paediatricians, 7.4 as gynaecologists, 6.7% as psychiatrists 3% as anaesthesiologists and 5.4% as others. In this study we focused on general practitioners, internists, surgeons, and psychiatrists. All other specialists were pooled as “different”. This allowed us to define specialists by the nature of their interactions with patients, in term of duration, intimacy, and mental versus bodily therapy.

Most of the respondents were employed by the public sector, representing the actual situation in Israel. There were 49.5% employed by public hospitals, 26.9% by Israeli HMOs (Health Maintenance Organizations), and only 7% practiced in the private sector. 8.5% of the respondents were retired doctors and ~ 1% military physicians. In this study we have pooled physicians in the private sector, in the army, retired ones and all other defining them as “different”. This allowed us to focus on the difference between physicians working in hospitals and those working at an HMO, assuming the former encounter more patients in severe/critical conditions, and therefore their attitudes to TFT and DAD are expected to be different.

A comparison of the survey population to that of the IMA population (Table 1) indicates underrepresentation of female physicians as well as younger doctors (ages 24–25) in our survey (at the expense of those aged 55–64).

### Attitudes towards telling the truth in cases of a poor prognosis

Respondents were asked to state their attitude towards telling a patient the full truth about a poor prognosis for his/her condition. Respondents could choose between support for the duty to tell the full truth (STFT), and opposition to the duty to tell the full truth (OTFT). The third option was to indicate an inability to choose between support and objection (see phrasing of question and answer options in Methods).

The question was answered by 2890 of the 2926 respondents (only 36 refrained from answering). There were 1374 in support of TFT, 1295 opposed it, and only 221 could not decide. When equated to the IMA population, support for TFT amounted to 47.4%, OTFT amounted to 45.7% and indecisiveness amounted to 6.9%.

The dissection of TFT supporters by their personal/professional characteristics is provided in the second column of Table 2 (a full analysis of all response options is provided in Additional file 1: Table S1 of the supplement). Male physicians were more inclined to support TFT than female physicians. Age was found to have a notable effect on attitudes towards truth-telling: Support of TFT was the highest among respondents aged 24–34, and the lowest among respondents aged 65 and over, with a clear age-dependent downward gradient in support between these two age groups (Table 2).

**Table 2:** Support of truth telling, doctor assisted death and their combination among different subgroups in the survey population

Respondent characteristics	Support telling full truth the (STFT)		Support doctor assisted death (SDAD)		Support assisted Death + Truth Telling (STFT + SDAD)	
	No (%) (b)	P (c)	No (%) (b)	P c)	No (%) (b)	P (C)
All respondents (*n* = 2926)(a)	1366 (47.6)		1586 (55.2)		760 (26.5)	
Gender		0.0003		0.0846		0.0427
Male (*n* = 1882)	931 (50.1)		1008 (54.0)		515 (27.9)	
Female (*n* = 1021)	435 (43.1)		578 (57.4)		243 (24.3)	
Age		<.0001		0.4338		.0001>
24–34 (*n* = 315)	185 (58.9)		174 (55.6)		107 (34.3)	
35–44(*n* = 570)	305 (54.0)		327 (57.7)		171 (30.4)	
45–54 (*n* = 551)	261 (47.8)		315 (58.2)		151 (28.1)	
55–64 (*n* = 711)	302 (43.2)		369 (52.5)		158 (22.8)	
65+ (*n* = 600)	234 (39.3)		337 (56.5)		141 (23.8)	
Medical Schooling		<.0001		.0001>		0.0659
Israel (*n* = 1475)	641 (43.9)		900 (61.5)		414 (28.5)	
Abroad (*n* = 720)	383 (53.7)		320 (44.9)		171 (24.2)	
Combined (*n* = 694)	341 (49.7)		361 (52.9)		171 (25.3)	
Position		0.0002		.0001>		0.0213
Interns (*n* = 468)	261 (56.2)		265 (57.0)		142 (30.7)	
Specialists (*n* = 1298)	582 (45.3)		681 (53.3)		305 (24.0)	
S. specialists (*n* = 872)	392 (45.6)		521 (60.1)		242 (28.3)	
Other (*n* = 241)	122 (51.0)		106 (44.2)		61 (25.5)	
Employment		0.0167		.0001>		0.0016
HMO (*n* = 852)	403 (48.1)		403 (47.9)		190 (23.0)	
Hospitals (*n* = 1425)	700 (49.5)		837 (59.3)		415 (29.6)	
Other(*n* = 605) (d)	256 (42.6)		338 (56.2)		149 (24.8)	
Specialization		0.0001		.0001>		.0001>
GP (*n* = 406)	166 (41.4)		187 (47.1)		68 (17.3)	
Internist (*n* = 810)	385 (48.1)		468 (58.3)		222 (27.9)	
Surgeons(*n* = 610)	310 (51.2)		347 (57.5)		171 (28.4)	
Psychiatrist (*n* = 191)	66 (35.1)		82 (43.2)		28 (15.0)	
Different (*n* = 836) (e)	414 (50.1)		447 (57.4)		252 (30.6)	

a) Numbers indicate representation of subgroup in the survey. Not all questions related to characteristics were answered by all surveyees, information amounted to 50–150

b) Number in parenthesis represent percentage out surveyees responding to the specific question

c) *P* value for indicated variable as compared to the rest of the population study

(d) Includes private clinics andprivate hospitals, military and retired physician

(e) Includes pediatricians, gynecologists, anesthetists and others (a)xxxx

Another notable effect on the attitude to TFT relates to the medical schooling of the respondents: Education abroad is associated with a notably higher tendency to support TFT than education in Israel. Interestingly, respondents who experienced combined education, namely partial education abroad and partial education in Israel exhibited intermediate tendencies (Table 2).

The place of employment, namely, Health Maintenance Organization (HMO) clinics versus public hospitals had no effect on attitude towards truth-telling. However, the field of specialization appeared to have some effect. Internists, surgeons and other specialists (including gynecologists and pediatricians; not shown) were similar in their support of TFT. A notable difference was observed when all these were compared to GPs and psychiatrists. GPs were less inclined to support TFT than other specialists (Table 2). The highest tendency to oppose TFT was observed among psychiatrists.

Weighted multivariate analyses (Table 3, upper rows) underline the effect of gender and age on supporting full truth-telling. Females were 0.72 fold less inclined than males to support TFT, and physicians in the age groups of 45–54, 55–64 and 65+ were less likely (0.70, 0.57 and 0.41 folds respectively) to support TFT than those aged 35–44. The effect of position on truth-telling is lost in the multivariate analysis suggesting that the effect observed in the bivariate analysis is probably attributed to age and reflects the fact that interns are younger than specialists.

**Table 3:** Weighted multinomial logistic regression analysis of the interrelationship between attitudes and socio demographic characteristics

Attitude	Variable	Effect	OD ratio	95% ConfidenceLimits	*P* value
Support truth telling (a)	Gender	Female vs. male	0.722	0.624–0.837	<.0001
	Age	24 to 34 vs. 35 to 44 45 to 54 vs. 35 to 44 55 to 64 vs. 35 to 44 65 plus vs. 35 to 44	1.221 0.707 0.572 0.409	0.915–1.629 0.592–0.845 0.483–0.676 0.337–0.496	0.1753 0.0001 <.0001 <.0001
	Medical education	Abroad vs. Israel Combined vs. Israel	1.351 1.374	1.165–1.564 1.188–1.589	<.0001 <.0001
	Specialization	Surgeons vs. Internists GP vs. Internist Psychiatry vs. Internist Different vs. Internist	1.139 0.729 0.666 1.178	0.987–1.313 0.564–0.943 1.050–0.423 0.993–1.398	0.0740 0.0016 0.0803 0.0598
	Gender	Male vs. Female	1.218	1.050–1.412	0.0093
Support doctor assisted death (a)	Medical education	Abroad vs. Israel Combined vs. Israel	0.564 0.800	0.487–0.654 0.692–0.926	<.0001 0.0028
	Workplace	Hospital vs. HMO Other vs. HMO	1.485 1.637	1.267–1.740 1.355–1.978	<.0001 <.0001
	Specialization	Surgeons vs. Internists GP vs. Internist Psychiatry vs. Internist Different vs. Internist	1.073 0.903 0.528 1.073	0.930–1.239 0.701–1.165 0.340–0.818 0.902–1.275	0.3357 0.4333 0.0043 0.4266
Support truth telling+doctor assisted death (a)	Gender	Female vs. male	0.815	0.692–0.960	0.0145
	Age	24 to 34 vs. 35 to 44 45 to 54 vs. 35 to 44 55 to 64 vs. 35 to 44 65 plus vs. 35 to 44	1.367 0.810 0.672 0.568	0.988–1.893 0.661–0.993 0.554–0.815 0.464–0.695	0.0594 0.0424 <.0001 <.0001
	Specialization	Surgeons vs. Internists GP vs. Internist Psychiatry vs. Internist Different vs. Internist	1.135 0.633 0.573 1.135	0.970–1.326 0.468–0.855 0.320–1.024 0.970–1.328	0.1132 0.0029 0.0602 0.0441

a: Variables included in the regression analysis: gender, age, medical schooling, position, employment specialization

The multivariate analysis confirms the effect of education: those educated abroad are 1.35 fold more likely to support TFT. The apparent effect of medical specialization is less obvious, the tendency of GPs to be less supportive of TFT is not significant when subjected to multivariate analysis. The tendency of psychiatrists to be less supportive is maintained (Table 2).

Taken together, all this suggests that Israeli physicians are divided in their attitude to TFT, and that support of TFT is dependent on the physicians’ personal traits (gender, age, formative medical education), and less so on their professional experience.

### Attitudes towards doctor-assisted life termination

Respondents were asked to indicate their willingness to take part in doctor-assisted death, given that this is requested by the patient and given that the medical conditions justify it. Respondents could choose between support for doctor-assisted death (SDAD), and opposition to doctor-assisted death (OTFT). Again, the third option was to indicate the inability to choose between support and objection (see phrasing of.

The question was answered by 2892 of the 2926 respondents (only 34 refrained from answering). Out of these, 1594 supported DAD, while 906 objected to DAD, and 392 of the respondents chose not express an opinion on this controversial subject. When equated to the IMA population, support for TFT amounted to 57.5%, objection to TFT amounted to 31.3% and indecisiveness amounted to 12.2%.

The dissection of respondents supporting doctor-assisted death (SDAD) by their personal/professional characteristics is provided in the fourth column of Table 2 (A full analysis of all response options is provided in Additional file 1: Table S2 of the supplement). In general, SDAD appears to depend on the personal characteristics of the respondents to a lesser extent than the support of TFT (Table 2). While female physicians were more inclined to support DAD than male physicians, age was not found to be a factor in the attitude towards DAD.

Medical education appears to have a notable effect on DAD support. Respondents who attended an Israeli medical school where more inclined to support DAD than those educated abroad. Respondents who experienced a combined education, in Israel and abroad, exhibited intermediate tendencies (Table 2).

The working environment had a significant effect on respondents’ attitude. Physicians employed by public hospitals were more supportive of DAD than those working for Israeli HMOs. Two groups of physicians stood out in their attitude to DAD. Both GPs and psychiatrist were less supportive of DAD than physicians with a different specialization.

The outcomes of weighted multivariate analyses (Table 3) were compatible with some of the above observations. The effect of gender was maintained, while the effect of specialization was less apparent. The effects of education and work environment were underlined. Respondents attending medical schools abroad were 0.564 times less inclined to support DAD than those educated in Israel. In addition, respondents working in a public hospital were 1.485 times more inclined to support DAD than those working for an HMO.

Taken together, a notable majority of Israeli physicians tended to support DAD. This support appears to be dependent mainly on the medical milieu: the formative milieu (education) and the professional environment (workplace).

### Interrelationships between attitudes to truth-telling and doctor-assisted death

In an attempt to examine the interrelationships between attitude to truth-telling and doctor-assisted death, we have crossed the responses to the two above mentioned queries: a) “Should the physician tell patients the full truth about their medical condition”; b) “Should a physician assist terminally ill patients in terminating their lives if medical conditions justify this”.

This yielded four combinations, enabling grouping the responders as follows:

1) Respondents who supported telling the full truth and supported doctor-assisted death – STFT/SDAD (weighted representation of 27.6%).

2) Respondents who supported telling the full truth but opposed doctor-assisted death – STFT/ODAD (weighted representation of 14.0%).

3) Respondents who opposed both, telling the full truth and doctor-assisted death – OTFT/ODAD (weighted representation of 14.5%).

4) Respondents who opposed telling the full truth yet supported doctor-assisted death – OTFT/SDAD (weighted representation of 26.3%).

Five hundred and forty-nine respondents could not be defined as belonging to these groups. Of these 329 formed an opinion on TFT, but were unable to form an opinion on DAD, and 161 formed an opinion on DAD, but were unable to form an opinion on TFTD. Only 59 respondents could not form an opinion on both TFT and DAD. This suggests that the rates of “recurrent” inability to decide are low, and that the choice of the response option “I am unable to choose” represents a genuine ethical statement. Interestingly only 10 respondents did not provide an answer to both queries.

Understanding the rationales behind the four patterns of response, STFT/SDAD, STFT/ODAD, OTFT/ODAD and OTFT/SDAD requires a separate in-depth study. Nevertheless, one could assume that respondents belonging to each one of these groups are guided by different ethical approaches. Thus, one can assume that a combination of support for TFT and DAD (STFT/SDAD) reflects respect for patient autonomy, identifying such responders as “Autonomists”. On the other hand, respondents who support TFT yet oppose DAD (STFT/ODAD) may be defined as “Deontologists”, who believe in “don’t lie and don’t kill”.

Respondents who believe that unconditional truth-telling is not a requisite can be divided into two groups based on their attitude to assisted death. Respondents, who oppose both TFT and DAD OTFT/ODAD, can represent “Traditionalists”, who stick to the classical concepts of physicians’ duties. The other group, which opposes TFT and at the same time support DAD death (OTFT/SDAD), is more difficult to decipher. The attitudes expressed here may relate to pragmatic utilitarianism, accompanied by compassion. We have defined them as “Compassionate Pragmatics”.

Respondent belonging to these all 4 groups were analyzed for their personal and professional characteristics (Additional file 1: Tables S3 and S4 in the supplement). We chose to focus on respondents identified as “Autonomists” (Table 2 sixth column). It is our belief that these constitute the most interesting group since they represent adherence to the more recent concepts related to interactions between physicians and patients. It is interesting to note that indeed, the “autonomistic” approach remains highly associated with younger age (Table 2 sixth column, and Table 3). Respondents in the age group of 55–64 as well as 65+ were less supportive of TFT + DAD than those aged 35–44 (OR of 0.672 and 0.568 respectively, Table3). In addition, female physicians were less supportive of both TFT and DAD than male physicians (OR 0.815) (Table 3).

Another, interesting characteristics is the association of “autonomic” approaches to the medical experience of respondents. GPs were less inclined to support both TFT and DAD than other specialists (Table 2, sixth column). This observation is substantiated by the multivariate analysis (Table 3). GPs were 0.633 times less supportive both of TFT and DAD than internists. The apparent tendency of psychiatrists to be less supportive of the “autonomistic” approach is only marginally significant.

Taken together, these observations suggest that “autonomistic” approaches are more acceptable to younger physicians, and that GPs stand out in their “non-autonomistic” attitude. Similar dissections were conducted for the three other response groups, namely the “Deontologists”, the “Conservatives” and the “Compassionate Pragmatics” (Additional file 1: Table S3). For lack of space, we shall not elaborate on these. It is however interesting to note that the deontological approach is associated with education abroad, whereas the pragmatic approach is associated with education in Israel.

## Discussion

In this paper we examine two debatable medical dilemmas, that of telling a patient the full truth about a bad prognosis (TFT), and that of assisting a fatally ill patient in life termination (DAD). This analysis yielded a number of valuable observations: a) The major part of the Israeli medical community supports DAD, b) Israeli physicians are divided in their attitude to TFT c) Age has a major effect on TFT: younger physicians are more supportive of TFT than older ones. d) Physicians educated in Israel tend to be less supportive of TFT and less supportive of DAD, than those educated abroad e) GPs are less like to respect patients autonomy than other specialists, and f) A combination of attitudes to TFT and DAD can yield a conceptual ethical typology. Each of these findings deserves a separate discussion that is provided here.

### Significance of support for DAD in Israel

Our survey suggests that the majority (57.5%) of Israeli physicians support DAD, whereas only 31.3 are opposed to it. A comparison to similar surveys conducted in other countries is limited because of differences in methodologies, in the target populations, in framing, as well as inconsistency in wording. However, a large number of studies conducted in Europe, USA and New Zealand demonstrate that in countries where assistance to die is not legalized support for it is in most cases lower than 50%, [27, 31–34]. It should be noted that support for DAD is shifting. For instance, in a Medscape survey conducted among US physicians in 2016 [35], 57% of the respondents supported DAD, whereas in a Medscape survey conducted in 2010 only 46% supported DAD [36] Similarly, 55% support rates were observed in a very recent poll among UK physicians [37], compared to lower support (25–39%) in earlier studies [31].

Our findings indicate that the support for DAD in Israel is on the higher side of the support scale in countries where assisted death is not legalized. This attitude stands out in a country where assisted life termination is not permitted by law, and is contradictory to the formal ethical framework of the Israeli Medical Association. Moreover, this level of support is not trivial in a country where the leading Jewish religion bans intervention in life termination [38], and where the specters of the homicidal atrocities of the holocaust still have an imprint on end of life decisions [29].

### Significance of attitudes to TFT in Israel

When the Israeli medical community was asked to express an opinion on telling the full truth to a patient about a poor prognosis for his/her condition, the attitudes were divided. About half supported TFT, while the other half opposed it.

Comparing this observation to observations made in other studies is difficult. Numerous surveys have been conducted on disclosure of diagnosis and prognosis, but most of them were framed in the context of cancer and end of life situations [34, 39, 40]. Unfortunately, the need to reveal bad news is not always associated with cancer diagnosis and prognosis, and bothers ophthalmologist, nephrologists, Geneticists, psychiatrists and many other specialists. In this survey, we tried to be as general as possible by not specifying the clinical situation. Moreover, we used the terms “telling the full truth” rather than “disclose prognosis”, to stress the fact that this is a value loaded question.

The notable divide in the opinions of physicians, suggests the coexistence of two fundamental approaches towards truth in Israel, the more traditional approach which examines truth through the utilitarian lens, and the more dogmatic view which perceives truth as a value by itself. The fact that about half of the respondents have a relaxed attitude towards truth-telling stands out against the fact that the Patients’ Rights Act of 1996 specifically states that Israeli physicians are required to tell patients the truth about their medical condition, and that the ethical guidelines of the IMA underline this requirement. Moreover, one would expect that after 30 years of teaching on the prominence of patient autonomy as a principle in medical ethics [1], support for truthful information is expected to be higher.

The observation that a substantial number of Israeli physicians support DAD, which is forbidden by law, yet oppose TFT, which is encouraged by guidelines, suggests a certain air of defiance. One could claim that the attitudes towards TFT and DAD coincide with the “Israeli spirit”, which is characterized by pragmatism, skepticism and flexible thinking [41].

### The effect of age on TFT and DAD

Age was found to have a very significant effect on truth-telling: younger Israeli physicians were more supportive of truth-telling than the older ones. Similar effects were observed in a survey assessing the attitudes of physicians from seven other countries, indicating that physicians under the age of 40 exhibited a higher proactive approach to discussing a poor prognosis [39]. The effect of age can be explained by the life cycle effect (age by itself), the period effect (the changing times) and the cohort effect (generational differences). One can claim that this is the effect of age: young less experienced physicians are more dogmatic towards truth, while older experienced physicians are more skeptic about the truth and tend to adopt paternalistic attitudes in their interactions with patients [42]. Alternately, one can also identify a simple period effect related to the Patients’ Rights Act of 1996 [43]. This law could have served as a turning point, and physicians who started practicing after 1966 are more inclined to tell the truth. The effect of the changing times on truth-telling can also be related to the increased attention to transparency in all aspects of modern life [44], and the increased emphasis given to autonomy and informed consent in health care [45]. Moreover, the tendency of lay individuals, nowadays, to learn about their medical situation through the internet and social media [3] gives rise to another dimension to truth-telling in the patient-physician interaction.

Another perspective for examining the tendency of young physicians to adhere to truth is the perspective of generational theory, which defines physicians born around 1980 belonging to the millennial cohort [46]. One could claim that increased support for TFT is related to specific attributes of the millennial physicians, differentiating them from physicians belonging to other generations (baby-boomers and X generation).Various studies have tried to attribute specific values to millennials, some of which could be relevant to the interactions between physicians and patients, and boost TFT. These include: placing a high importance on values, appreciation of individualism and willingness to cooperate with others [47–49].

The relative contribution of these various effects to TFT remains open and is worth clarifying, since it might provide a clue for predicting future directions in patient-physician interactions, and help understand the possible effect of such interactions on better outcomes for patients [50].

In contrast to the notable effect of age on attitude to TFT, all age groups supported DAD to the same extent. Surprisingly, none of the age related effects described above (life cycle effects, period effects and cohort effects) applies to attitudes towards assisting death. Moreover, our findings stand in contrast with studies from other countries indicating that younger physicians are more lenient towards support for assisted life termination [51]. It appears, therefore, that, in Israel matters related to life termination are not affected by tangential factors such as age.

### The effect of medical education on ethical perceptions

One of the characteristics of the medical community in Israel is the fact that about half of the practicing physicians have been educated in Israeli medical schools. Doctors educated abroad include those immigrating to Israel after graduating from medical schools in their native countries, and those travelling abroad from Israel to attend medical schools abroad (most often in Italy, Hungary Romania former USSR countries and Jordan). Our findings suggest that medical education appears to have a notable effect on formulating opinions about TFT and DAD. Those educated in Israel appear to be less supportive of TFT and more supportive of DAD than those educated abroad.

The diversity of the medical schools attended by Israelis is rather high, and precludes a comprehensive assessment of the ethical education provided to their students. On the other hand, all the 4 more established medical schools in Israel provide modular ethical teaching through specific courses offered during the three premedical years. In addition, in all Israeli schools the “bed side” clinical education in the hospital starts as early as the 3rd or 4th year of schooling. This could result in a situation where the academic ethical concepts learned in class are immediately affected by the reality of hospital life, and by the interactions with older doctors. The outcome of this could be the promotion of utilitarian and compassionate pragmatism. In contrast, students who attended medical schools abroad probably acquired tendencies that are more deontological in nature. All this correlates well with the assumption that formal ethical education is not an isolated factor, but part of a web of interrelated factors that influence educational outcomes [52].

The contribution of the real medical environment in the formation of ethical attitudes towards TFT and DAD is substantiated by two observations: a) Combined education, which practically means pre-clinical years abroad and clinical years in Israel, leads to intermediate levels of support for TFT (higher than for Israeli education, yet lower than for education abroad), as well as intermediate levels of support of DAD. b) Cross tabulation of age with place of education indicates that the effect of education on TFT, is pronounced among young physicians (65% for those educated abroad versus 50% for those educated in Israel). Yet, this difference becomes less pronounced (45% for those educated abroad and 40% for those educated in Israel) among older physicians, suggesting that the imprint of the medical school wanes with time.

### The effect of specialization and the work place

Many studies on DAD have often explored the attitude of physicians who are confronted by terminal suffering. Some studies have found that specialists such as oncologists, geriatrics and those involved in palliative care have more negative attitudes towards DAD [51, 53, 54]. In our study, we attempted to rather focus on the nature of the interaction between patient and physicians of different specializations, believing that this might be relevant both to TFT and DAD. We have compared the attitudes of GPs, internists and surgeons, assuming that this will allow representation of a gradient of intimacy and duration of interactions. We also compared these specialists to psychiatrists, assuming that this will exemplify interactions that relate to mental aspects rather than bodily aspects of therapy.

Our findings indeed suggest that the nature of patient-physician interaction may play a role in perceptions of TFT and DAD. Psychiatrists stand out in their attitude by exhibiting much lower support for both TFT and DAD (Tables 2, 3). While, this could be related to limited professional contact with fatal morbidities, one should not forget that certain mental disorders entail tremendous suffering and can affect life expectancy. One should note that the discourse on DAD in the context of severe psychiatric cases [55] is still highly controversial, and psychiatrists’ attitudes may change in the future. The low support of psychiatrists for truth-telling remains puzzling, and could be related to the still prevailing belief that truth can be anti-therapeutic in psychiatry [56].

Surgeons can be perceived as physicians who relate mainly to the physical aspects of medical practice. They “repair the body” and their interactions with patients are restricted in duration and in the level of intimacy [57]. Interestingly, the attitude of surgeons to both DAD and TFT did not differ from that of internists nor from that of other specialists.

In contrast, the specific aspects of the practice of GPs, who are supposed to have long and intimate interactions with patients, appear to affect some of their ethical stands. Contrary to intuition, GPs are less supportive of TFT than other specialists, suggesting that close interactions with the patients serve as a barrier, when it comes to truth-telling. This could relate to the emotional toll on physicians that comes with the need to face a patient and disclose bad news [58]. Interestingly the effect of GP practice has a lesser impact on their attitude to DAD (Table 3) .

All the above observations can be interpreted not only in the context of patient-physician interactions, but also in the context of different exposures to life threatening morbidities. In an attempt to dissect between the contribution of a physician’s medical experience and the nature of the interaction with the patient, we have cross tabulated specialization with the workplace of physicians. This allowed us to focus on physicians of different specialties all employed by HMOs and therefore less exposed to terminal situations. While 48.4 and 48.6% of HMO–employed physicians and internists supported TFT, only 42.6% of GPs did so, supporting the assumption that in the case of GPs higher acquaintance with the patient is indeed a barrier to truth-telling. The differences in attitude towards DAD were less pronounced: 45, 47 and 52% of HMO-employed GPs, surgeons or internists supported DAD, respectively.

A comparison between surgeons employed by hospitals and surgeons employed by HMOs did not reveal a difference in attitude to TFT, but did reveal a notable difference in attitude to DAD: support for DAD was 61% among surgeons practicing in hospitals as opposed to 47.5% among those practicing in an HMO. This observation suggests that higher exposure to end of life situations enhances the understanding that in certain cases, life termination can be legitimate.

Taken together, the effects of specialization on attitudes to TFT and DAD suggest interplay between the nature of the interactions with patients on one hand and the medical experiences accumulated by physicians on the other hand. The effect of closeness appears to be more relevant to TFT, and the effect of exposure more relevant to DAD.

### Typology based on the interrelationship between attitudes to TFT and DAD

Matters as complex as DAD and TFT can be approached by using various typologies. Typology can be based on clinical situations, where segmentation of attitudes to both DAD and TFT are base on the severity of the patient’s situation. Typology can be based on the definition of the mode of assistance for life termination ranging from the cessation of life sustaining measures to the administration of lethal drugs. Topology can also be based on variations in the interpretation of truth and deception. Some of these typologies have been used successfully by others to portray the complexity of interactions between patients and physicians in challenging situations [33, 59, 60].

Typology based on conceptual ethical characterization [43] has been used less frequently to examine end of life decisions (Fin [61]). In this study we used a typology based on a combination of attitudes to DAD and TFT, in sn attempt to gain some conceptual characterization. To this end, we have defined four attitudinal variants. Physicians were typified as: a) autonomists (supporters of DAD and TFT), b) compassionate pragmatists (supporters of DAD and objectors of TFT), c) deontologists (objectors to DAD and supporters of TFT), and d) traditionalists (objectors to both DAD and TFT).

All four attitudinal approaches appear to be well represented in the Israeli medical community, yet the autonomists and compassionate pragmatists (each exhibiting about 25% representation) appear be more prevalent than the deontologists and traditionalists (each exhibiting about 15% representation). This implies a co-dominance of two ethical approaches, which are essentially different and even contradicting. Interestingly, the personal /professional characteristics of the physicians that adopt these two approaches are different (Additional file 1: Table S3 and S4). The autonomic approach is more prevalent than the pragmatic approach among young millennial physicians, whereas compassionate pragmatic approaches are more prevalent among female physicians.

Future tendencies are hard to predict. The understanding that millennials will dominate the medical community suggests a shift towards autonomic approaches, yet the foreseen increase in the proportion of female physicians in the medical workforce [62] could suggest a shift towards pragmatic compassion.

### Limitation

The 10% response rate attained in our survey is considered as low and is an outcome of the methodology that we have used. We chose to address the entire population of the Israeli Medical Association members, even though previous experience indicated that this could result in a low response rate. This gave us access to most of the practicing physicians in the country, without any screening or pre-selection, and allowed us to attain a survey group of 2926 respondents. In addition, this provided good representation of the various sub-populations, relevant to our study allowing meaningful statistically analyses. The analyses were weighted to the actual distribution of gender, age and specialization in the Israeli medical community, but this did not make a notable difference. It should be noted that the traditional perceptions on the desired response rates in surveys have been challenged in recent years. Evaluations of national surveys [63] with response rates ranging from 5 to 54% have indicated that studies with a much lower response were often only marginally less accurate than those with much higher response rates.

The other limitation of this paper is the lack of any analysis related to cultural variants. Religion and religiosity were found to affect attitudes towards DAD in studies conduct in different countries [64] as well as in Israel [30, 65]. The methodology that we have used led us to decide against asking respondents to relate to religion or degree of religiosity. We felt that in times when the schisms between religious and secular Israelis is at one of its peaks, it would be inappropriate to ask about religion in a formal questionnaire distributed by the Israeli Medical Association.

The lack of information on the representation of religious respondents in our survey population led us to conduct a rough sensitivity analysis. To this end we made the assumption that the representation of religious physicians in the survey population is only 10%, which is only half of the proportion of religious Jews (not ultra-religious) in the Israeli population. In addition we assumed that religious physicians will always oppose DAD. Under these stringent conditions, the support for DAD in our study is expected to drop only by 5%.

## Conclusions

The high support for doctor-assisted death among Israeli physicians may reflect attitudinal changes, but does not predict necessary forthcoming changes in legislation and policy. Moreover, while this observation supports the understanding that the debate over doctor-assisted death in developed countries is not dying out, it does not necessarily indicate that the Dutch/Belgian approach to life termination will be adopted worldwide.

The split between those supporting truth-telling to patients and those opposing it could indicate a state of transition between old tendencies and new tendencies. Nevertheless, this situation may have deleterious effects on the trust in the medical system, and attempts should be made to formulate uniform guidelines.

The fact that younger physicians are more inclined to adopt attitudes compatible with respect for patient autonomy could imply a generational shift and should be met with optimism, yet the tendency of general practitioners to be less respectful of autonomy is worrisome.

The notable attitudinal difference between physicians attending medical schools in Israel and those attending schools broad suggests that formal ethical education combined with local influences and national characteristics can forge the ethical profile of a given medical community.

Typology based on attitudes to assisted death and truth-telling, can be developed into a generic tool for probing the medico-ethical atmosphere in different countries at different time points.

## Additional file

Additional file 1: **Table S1.** Attitude towards truth telling among different subgroups in the survey population. **Table S2.** Attitude towards doctor assisted death among different subgroups in the survey population**. Table S3.** Combined attitude towards doctor assisted death and truth telling among different subgroups in the survey population. **Table S4.** Non-weighted multinomial logistic regression analysis of the interrelationship between combined attitudes and socio demographic characteristics. (DOCX 35 kb)

## Abbreviations

DAD: Doctor-assisted death
IMA: Israeli Medical Association
ODAD: Objection to DAD
OTFT: Objection to TFT
SDAD: Support of DAD
STFT: Support of TFT
TFT: Telling full truth
