# Supplementary material for: Truth-telling and doctor-assisted death as perceived by Israeli physicians
Source: BMC Med Ethics. 2019 Feb 18;20:13. doi: 10.1186/s12910-019-0350-5 (PMC6380017; doi:10.1186/s12910-019-0350-5)
Supplement: Supplementary file 1 — Table S1. Attitude towards truth telling among different subgroups in the survey population. Table S2. Attitude towards doctor assisted death among different subgroups in the survey population. Table S3. Combined attitude towards doctor assisted death and truth telling among different subgroups in the survey population. Table S4. Non-weighted multinomial logistic regression analysis of the interrelationship between combined attitudes and socio demographic characteristics. (DOCX 35 kb) [file 12910_2019_350_MOESM1_ESM.docx]

| **P value**  Chi-Square | **No position**  No (%) | **Oppose telling full truth the**  **(OTFT)**  No (%) | **Support telling full truth the**  **(STFT)**  No (%) | **Respondent characteristics** |
| --- | --- | --- | --- | --- |
|  | 221**(7.7)** | 1283**(44.7)** | 1366 **(47.6)** | **All respondents** (n=2926) |
| <.0001 |  |  |  | **Gender** |
|  | 99**(5.3)** | 830 **(44.6)** | 931 **(50.1)** | Male (n=1882) |
|  | 112 **(12.1)** | 453 (**44.8)** | 435 **(43.1)** | Female (n=1021) |
| <.0001 |  |  |  | **Age** |
|  | 27 **(8.7)** | 102**(32.3)** | 185**(59.0)** | 24-34 (n=315) |
|  | 62 **(11.0)** | 198**(35.0)** | 305**(54.0)** | 35-44 (n=570) |
|  | 40 **(7.3)** | 245 **(44.9)** | 261 **(47.8)** | 45-54 (n=551) |
|  | 56 **(8.0)** | 341 **(48.8)** | 302 **(43.2)** | 55-64 (n=711) |
|  | 21 **(3.5)** | 340**(57.2)** | 234 **(39.3)** | 65+ (n=600) |
|  | 49.2+12.7 | 55.4+13.7 | 50.7+13.9 | Mean age 52.76+14.0 |
| 0.0001 |  |  |  | **Medical Schooling** |
|  | 131**(9.2)** | 689**(47.1)** | 641 **(43.9)** | Israel (n=1475) |
|  | 44**(6.2)** | 286**(40.1)** | 383 **(53.7)** | Abroad (n=720) |
|  | 42**(6.1)** | 303**(44.2)** | 341 **(49.7)** | Combined (n=694) |
| 0.0005 |  |  |  | **Position** |
|  | 35 **(7.5)** | 168 **(36.2)** | 261 **(56.3)** | Intern (n=468) |
|  | 108 **(8.4)** | 596 **(46.3)** | 583 **(45.3)** | Specialist (n=1298) |
|  | 54 **(6.3)** | 414 **(48.1)** | 392 **(45.6)** | S. specialist (n=872) |
|  | 18 **(7.5)** | 99 **(41.4)** | 122 **(51.1)** | Other (n=241) |
| 0.0028 |  |  |  | **Employment** |
|  | 79 **(9.4)** | 355**(42.4)** | 403 **(48.2)** | HMO (n=852) |
|  | 100 **(7.1)** | 614 **(43.4)** | 700 **(49.5)** | Hospitals (n=1425) |
|  | 39**(6.5)** | 306 **(50.9)** | 256 **(42.6)** | Other (n=605) |
| <.0001 |  |  |  | **Specialization** |
|  | 39**(9.7)** | 196 **(49.0)** | 166 **(41.4)** | GP (n=406) |
|  | 58**(7.2)** | 357 **(44.6)** | 385 **(48.1)** | Internist (n=810) |
|  | 35**(5.8)** | 260 **(43.0)** | 310 **(51.2)** | Surgeon (n=610) |
|  | 26**(13.8)** | 98 **(51.1)** | 66 **(35.1)** | Psychiatry (n=191) |
|  | 58 **(7.2)** | 352 **(42.6)** | 414 **(50.1)** | Different (n=836) |

**Table S1: Attitude towards truth telling among different subgroups in the survey population.**

| **P value**  Chi-Square | **No position**  No (%) | **Oppose doctor assisted death**  **(ODAD)**  No (%) | **Support doctor assisted death**  **(SDAD)**  No (%) | **Respondent characteristics** |
| --- | --- | --- | --- | --- |
|  | 390**(13.6)** | 896**(31.2)** | 1586 (55.2) | **All respondents** (n=2926) |
| 0.0001 |  |  |  | **Gender** |
|  | 229**(12.3)** | 628**(33.7)** | 1008 **(54.0)** | Male (n=1882) |
|  | 161 **(16.0)** | 268 (**26.6)** | 578 **(57.4)** | Female (n=1021) |
| 0.407 |  |  |  | **Age** |
|  | 36 **(11.6)** | 103 **(32.8)** | 174 **(55.6)** | 24-34 (n=315) |
|  | 85 **(15.0)** | 155 **(27.3)** | 327 **(57.7)** | 35-44(n=570) |
|  | 76 **(14.0)** | 150 **(27.8)** | 315 **(58.2)** | 45-54 (n=551) |
|  | 90 **(12.8)** | 244 **(34.7)** | 369 **(52.5)** | 55-64 (n=711) |
|  | 74 **(12.4)** | 186 **(31.1)** | 337 **(56.5)** | 65+ (n=600) |
|  | 52.2+13.3 | 53.1+14.2 | 52.7+14.0 | Mean age 52.76+14.0 |
| <.0001 |  |  |  | **Medical Schooling** |
|  | 177 **(12.1)** | 387 **(26.4)** | 900**(61.5)** | Israel (n=1475) |
|  | 108**(15.1)** | 285 **(40.0)** | 320**(44.9)** | Abroad (n=720) |
|  | 101**(14.8)** | 221 **(32.4)** | 361 **(52.9)** | Combined (n=694) |
| 0.0006 |  |  |  | **Position** |
|  | 56 **(12.0)** | 144 **(31.0)** | 265 **(57.0)** | Intern (n=468) |
|  | 184 **14.4)** | 413 **(32.3)** | 681 **(52.3)** | Specialist (n=1298) |
|  | 107 **(12.3)** | 239 **(27.6)** | 521 **(60.1)** | S. specialist (n=872) |
|  | 37 **(15.4)** | 97 **(40.4)** | 106 **(44.2)** | Other (n=241) |
| <.0001 |  |  |  | **Employment** |
|  | 134 **(15.9)** | 304**(36.2)** | 403 **(47.9)** | HMO (n=852) |
|  | 172 **(12.2)** | 403 **(28.5)** | 837**(59.3)** | Hospitals (n=1425) |
|  | 78**(13.0)** | 185 **(30.8)** | 338 **(56.2)** | Other(n=605) |
| <.0001 |  |  |  | **Specialization** |
|  | 53**(13.3)** | 157 **(39.6)** | 187 **(47.1)** | GP (n=406) |
|  | 87**(10.9)** | 247 **(30.8)** | 468 **(58.3)** | Internist (n=810) |
|  | 86**(14.2)** | 171**(28.3)** | 347 **(57.5)** | Surgeon (n=610) |
|  | 38**(20.0)** | 70 **(36.8)** | 82 **(43.2)** | Psychiatry (n=191) |
|  | 117 **(14.1)** | 237 **(28.5)** | 447**(57.4)** | Different (n=836) |

**Table S2 : Attitude towards doctor assisted death among different subgroups in the survey population**

| **ODAD+OTFT**  Classists  No (%) | **ODAD+STFT**  (Deontologists)  No (%) | **SDAD+OTFT**  (Pragmatic)  No (%) | **SDAD+STFT**  (Autonomist)  No (%) |  |
| --- | --- | --- | --- | --- |
| 412 **(14.5)** | 419 **(14.4)** | 710 **(25.0)** | 758 **(26.6)** | **All respondents** (n=2926) |
| 0.0022 | 0.0013 | 0.0563 | 0.0427 | **Gender**  *P value* |
| 295 **(16.0)** | 301 **(16.3)** | 440 **(23.8)** | 515  **(27.9)** | Male (n=1882) |
| 177 **(11.7)** | 118 **(11.8)** | 270 (**27.1)** | 243 **(24.3)** | Female (n=1021) |
| 0.0022 | 0.0083 | <.0001 | <.0001 | **Age**  *P value* |
| 37 **(11.9)** | 57 **(18.3)** | 53 **(16.9)** | 107 **(34.3)** | 24-34 (n=315) |
| 55 **(9.68)** | 89 **(15.8)** | 115 **(20.4)** | 171 **(30.4)** | 35-44(n=570) |
| 66 **(12.3)** | 69 **(12.9)** | 145 **(27.0** | 151 **(28.1)** | 45-54 (n=551) |
| 123 **(17.8)** | 101 **(14.6)** | 185 (**26.6)** | 158 **(22.8)** | 55-64 (n=711) |
| 113 **(19.10)** | 68 **(115)** | 182 (**30.7)** | 141 **(23.8)** | 65+ (n=600) |
| 0.5289 | <.0001 | <.0001 | 0.0659 | **Schooling**  *P value* |
| 202 **(13.9)** | 154 **(10.6)** | 410 **(28.2)** | 414 **(28.5)** | Israel (n=1475) |
| 111 **(15.7)** | 154 **(21.8)** | 135 (**19.1)** | 171 **(24.2)** | Abroad (n=720) |
| 96 **(14.8)** | 113 **(16.7)** | 165 (**23.2)** | 171 **(25.3)** | Combined (n=694) |
| 0.3820 | 0.0078 | 0.0051 | 0.0016 | **Employment**  *P value* |
| 120 **(14.5)** | 149 **(18.0)** | 176 **(21.3)** | 190 **(23.0)** | HMO (n=852) |
| 191 **(13.6)** | 192 **(13.7)** | 363 (**25.9)** | 415 **(29.4)** | Hospitals (n=1425 |
| 97 **(16.2)** | 78 **(13.0)** | 171 (**28.5)** | 149 **(24.8)** | Other(n=605) |
| 0.0022 | 0.3423 | 0.6777 | <.0001 | **Specialization**  *P value* |
| 74 **(18.9)** | 69 **(17.6)** | 96 **(24.5)** | 68 **(17.3)** | GP (n=406) |
| 113 **(14.2)** | 118 **(14.8)** | 207 (**26.0)** | 222 **(27.9)** | Internist(n=810) |
| 70 **(11.7)** | 93 **(15.5)** | 159 **(26.9)** | 171 **(28.4)** | Surgeon(n=610) |
| 38 **(20.3)** | 23 **(12.3)** | 41 (**21.9)** | 28 **(15.0)** | Psychiatry(n=191) |
| 107 **(13.0)** | 112 **(13.6)** | 200 **(24.3)** | 252 **(30.61)** | Different (n=836) |

**TableS3 : Combined attitude towards doctor assisted death and truth telling among different subgroups in the survey population**

| **Pr>ChiSq** | **95% Confidence Limits** | **OD ratio** | **Effect** | **Variable** | **Attitude** |
| --- | --- | --- | --- | --- | --- |
| 0.0466 | 0.676 - 0.997 | 0.821 | Female vs. male | **Gender** | **Support truth telling**  **+**  **Support doctor**  **assisted death**  Autonomist |
| 0.0016  0.0023 | 0.509 - 0.854  0.499 - 0.859 | 0.659  0.655 | 55 to 64 vs. 35 to 44  65 plus vs. 35 to 44 | **Age** |  |
| 0.8367  0.0006  0.0018  0.1417 | 0.802 – 1.314  0.418 - 0.787  0.318 - 0.771  0.946 - 1.471 | 1.026  0.573  0.495  1.180 | Surgeons vs. Internists  GP vs. Internist  Psychiatry vs. Internist  Different vs. Internist | **Specialization** |  |
| 0.0019 | 0.863 - 0.522 | 0.671 | Female vs. male | **Gender** | **Support truth telling**  **+**  **Reject Doctor**  **assisted death**  Deontologists |
| 0.0012 | 0.345 - 0.769 | 0.515 | 65 plus vs. 35 to 44 | **Age** |  |
| <.0001  <.0001 | 1.720 - 2.770  1.349 – 2.404 | 2.111  1.801 | Abroad vs. Israel  Combined vs. Israel | **Medical education** |  |
| 0.0030  0.0411 | 0.500- 0.864  0493- 0.968 | 0.659  0.697 | Hospital vs. HMO  Other vs. HMO | **Employment** |  |
| 0.0025 | 1.110- 1.630 | 1.345 | Female vs. male | **Gender** | **Reject truth telling**  **+**  **Suport doctor**  **assisted death**  Utilitarian |
| 0.0015  0.0001  <.0001 | 1.211- 2.237  1.337- 2.419  1.631 -3.164 | 1.646  1.799  2.272 | 45 to 54 vs. 35 to 44  55 to 64 vs. 35 to 44  65 plus vs. 35 to 44 | **Age** |  |
| 0.0004  0.0313 | 0.510 - 0.822  0.620 - 0.978 | 0.647  0.779 | Abroad vs. Israel  Combined vs. Israel | **Medical education** |  |
| 0.0321 | 1.022- 1.629 | 1.290 | Hospital vs. HMO | **Employment** |  |
| 0.0005 | 1.155- 1.683 | 1.394 | Female vs. male | **Gender** | **Rejects truth telling**  **+**  **Rejects doctor**  **assisted death**  Classists |
| 0.0026  0.0005  <.0001 | 1.175- 2.151  1.250- 2.230  1.583 -2.881 | 1.590  1.670  2.136 | 45 to 54 vs. 35 to 44  55 to 64 vs. 35 to 44  65 plus vs. 35 to 44 | **Age** |  |

**TableS4 : Non-weighted multinomial logistic regression analysis of the interrelationship between combined attitudes and socio demographic characteristics**
